# Molecular surveillance for polymorphisms associated with artemisinin-based combination therapy resistance in *Plasmodium falciparum* isolates collected in Mozambique, 2018

**DOI:** 10.1186/s12936-021-03930-9

**Published:** 2021-10-12

**Authors:** Arlindo Chidimatembue, Samaly S. Svigel, Alfredo Mayor, Pedro Aíde, Abel Nhama, Lídia Nhamussua, Arsénio Nhacolo, Quique Bassat, Crizólgo Salvador, Sónia Enosse, Abuchahama Saifodine, Eva De Carvalho, Baltazar Candrinho, Rose Zulliger, Ira Goldman, Venkatachalam Udhayakumar, Naomi W. Lucchi, Eric S. Halsey, Eusébio Macete

**Affiliations:** 1 Centro de Investigação em Saúde de Manhiça (CISM), Maputo, Mozambique; 2 Malaria Branch, Division of Parasitic Diseases and Malaria, Centers for Disease Control and Prevention, Atlanta, GA USA; 3 ISGlobal, Hospital Clínic – Universitat de Barcelona, Barcelona, Spain; 4 Instituto Nacional de Saúde (INS), Ministério da Saúde, Maputo, Mozambique; 5 ICREA, Pg. Lluís Companys 23, 08010 Barcelona, Spain; 6 Pediatric Infectious Diseases Unit, Pediatrics Department, Hospital Sant Joan de Déu (University of Barcelona), Barcelona, Spain; 7 Consorcio de Investigación Biomédica en Red de Epidemiología Y Salud Pública (CIBERESP), Madrid, Spain; 8 United States President’s Malaria Initiative, US Agency for International Development, Maputo, Mozambique; 9 World Health Organization (WHO), Maputo, Mozambique; 10 National Malaria Control Programme, Ministry of Health, Maputo, Mozambique; 11 United States President’s Malaria Initiative, Centers for Disease Control and Prevention, Maputo, Mozambique; 12 United States President’s Malaria Initiative, Atlanta, GA USA; 13 National Directorate of Public Health, Ministry of Health, Maputo, Mozambique

**Keywords:** Antimalarial drug resistance, *Plasmodium falciparum*, *pfk13*, *Pfcrt*, *pfmdr1*, Artemisinin-based combination therapy (ACT), Polymorphisms, Mozambique

## Abstract

**Background:**

Due to the threat of emerging anti-malarial resistance, the World Health Organization recommends incorporating surveillance for molecular markers of anti-malarial resistance into routine therapeutic efficacy studies (TESs). In 2018, a TES of artemether-lumefantrine (AL) and artesunate-amodiaquine (ASAQ) was conducted in Mozambique, and the prevalence of polymorphisms in the *pfk13*, *pfcrt,* and *pfmdr1* genes associated with drug resistance was investigated.

**Methods:**

Children aged 6–59 months were enrolled in four study sites. Blood was collected and dried on filter paper from participants who developed fever within 28 days of initial malaria treatment. All samples were first screened for *Plasmodium falciparum* using a multiplex real-time PCR assay, and polymorphisms in the *pfk13*, *pfcrt,* and *pfmdr1* genes were investigated by Sanger sequencing.

**Results:**

No *pfk13* mutations, associated with artemisinin partial resistance, were observed. The only *pfcrt* haplotype observed was the wild type CVMNK (codons 72–76), associated with chloroquine sensitivity. Polymorphisms in *pfmdr1* were only observed at codon 184, with the mutant 184F in 43/109 (39.4%) of the samples, wild type Y184 in 42/109 (38.5%), and mixed 184F/Y in 24/109 (22.0%). All samples possessed N86 and D1246 at these two codons.

**Conclusion:**

In 2018, no markers of artemisinin resistance were documented. Molecular surveillance should continue to monitor the prevalence of these markers to inform decisions on malaria treatment in Mozambique.

## Background

Malaria remains a leading health problem in many countries, particularly those in tropical and subtropical regions of the world [1]. In Mozambique, malaria represents a major cause of morbidity and mortality, accounting for 29% of all deaths and approximately 42% of deaths among children less than 5 years of age [2]. *Plasmodium falciparum* is the predominant malaria parasite species in the country [3].

One of the fundamental steps toward malaria control is the rapid diagnosis and correct treatment of symptomatic cases with an effective anti-malarial [4]. Anti-malarial drug resistance continues to be a major hurdle to malaria control efforts in some settings. Since replacing chloroquine (CQ) with a combination of amodiaquine (AQ) + sulfadoxine-pyrimethamine (SP) for uncomplicated malaria treatment in 2003, the Mozambique national treatment guidelines have experienced various adjustments as parasites became resistant to treatments [5]. In 2006, artemisinin-based combination therapy (ACT) was formally introduced by adopting artesunate (AS) + SP as a first-line treatment for uncomplicated *P. falciparum* malaria [6, 7]. Subsequently, the last change occurred in 2009, when the country introduced artemether-lumefantrine (AL) and artesunate-amodiaquine (ASAQ) as the official first-line treatments, with ASAQ as a backup in situations when AL is contraindicated [6–8]. While ASAQ is part of the national treatment algorithm, there has been limited procurement and use. In artemisinin-based combinations, the artemisinin component is short-acting and kills the majority of parasites within the first 2 days of treatment; the remaining parasites are cleared by the longer-acting partner drug [9], thus helping to abate the acquisition of parasite resistance to the treatment. However, resistance to artemisinin derivatives, defined as delayed parasite clearance (presence of > 10% parasitaemia on day 3 after the start of treatment), has been reported in Southeast Asia [10–12] and Rwanda [13]. Resistance to specific anti-malarials is associated with polymorphisms, such as a single nucleotide polymorphisms (SNPs), a combination of SNPs, or gene copy number variation in drug target genes.

To monitor the efficacy of anti-malarial treatment, the World Health Organization (WHO) recommends periodic (at least every 2 years) monitoring of the first and second-line anti-malarial treatments [14] and, in addition, molecular surveillance of resistance markers is encouraged. Artemisinin partial resistance is associated with polymorphisms in the *P. falciparum kelch 13* (*pfk13*) gene [10] and ten SNPs in *pfk13* gene are currently validated molecular markers for artemisinin partial resistance: F446**I**, N458**Y**, M476**I**, Y493**H**, R539**T**, I543**T**, P553**L**, R561**H**, P574**L** and C580**Y** [14]. One of these mutations, R561**H**, has been reported to be present in multiple samples from different sites in Rwanda [13, 15, 16], highlighting the importance of conducting molecular surveillance to identify emerging artemisinin and partner drug resistance genotypes. To date, there have been no reports of *pfk13* polymorphisms associated with artemisinin partial resistance in Mozambique [17, 18].

Resistance to CQ is mainly associated with SNPs in the *P. falciparum chloroquine resistance transporter* (*pfcrt*) gene, resulting in an amino acid change from lysine (K76) to threonine (76**T**) at position 76; however, the *P. falciparum multi-drug resistance* (*pfmdr1*) gene may also play a role in CQ resistance [19, 20]. The most commonly reported *pfcrt* mutations are observed in codons 72, 74–76 [21]. The wild type CVMNK haplotype is associated with CQ sensitivity, while the CV**IET** and **S**VMN**T** haplotypes are associated with CQ resistance, with CV**IET** being the more common of the latter two haplotypes in Africa [22, 23].

The *pfmdr1* gene is implicated in lower sensitivity or tolerance to several anti-malarial drugs, including CQ, AQ, and lumefantrine [22, 24]. In Africa, the most relevant polymorphisms of *pfmdr1* include N86**Y**, Y184**F** and D1246**Y** [8, 25]. Mutations at positions S1034**C** and N1042**D** of *pfmdr1* are rarely reported on the continent [8, 26]. The 86**Y** mutation has been associated with decreased CQ and AQ sensitivity, while the N86 wild type codon has been implicated in decreased sensitivity to lumefantrine. The N86, 184**F**, and D1246 (N**F**D) haplotype is associated with decreased sensitivity to AL, while the 86Y, Y184, and 1246Y (YYY) haplotype is reported to be associated with decreased sensitivity to ASAQ [25]. In Mozambique, the prevalence of *pfmdr1* mutations was low in the capital city of Maputo, although the alleles N86 and 184**F** showed a significantly increased prevalence after the introduction of ACT [8].

Molecular surveillance for drug resistant parasites is part of a comprehensive approach along with TESs for early detection and subsequent prevention of spread of resistant parasites by permitting timely implementation of appropriate alternative treatment policy decisions. This study’s aim was to analyse the prevalence of molecular markers associated with *P. falciparum* resistance to anti-malarial drugs in the *pfk13*, *pfmdr1,* and *pfcrt* genes in samples collected during a 2018 TES in four sentinel sites in Mozambique.

## Methods

### Study sites

**Fig. 1:**
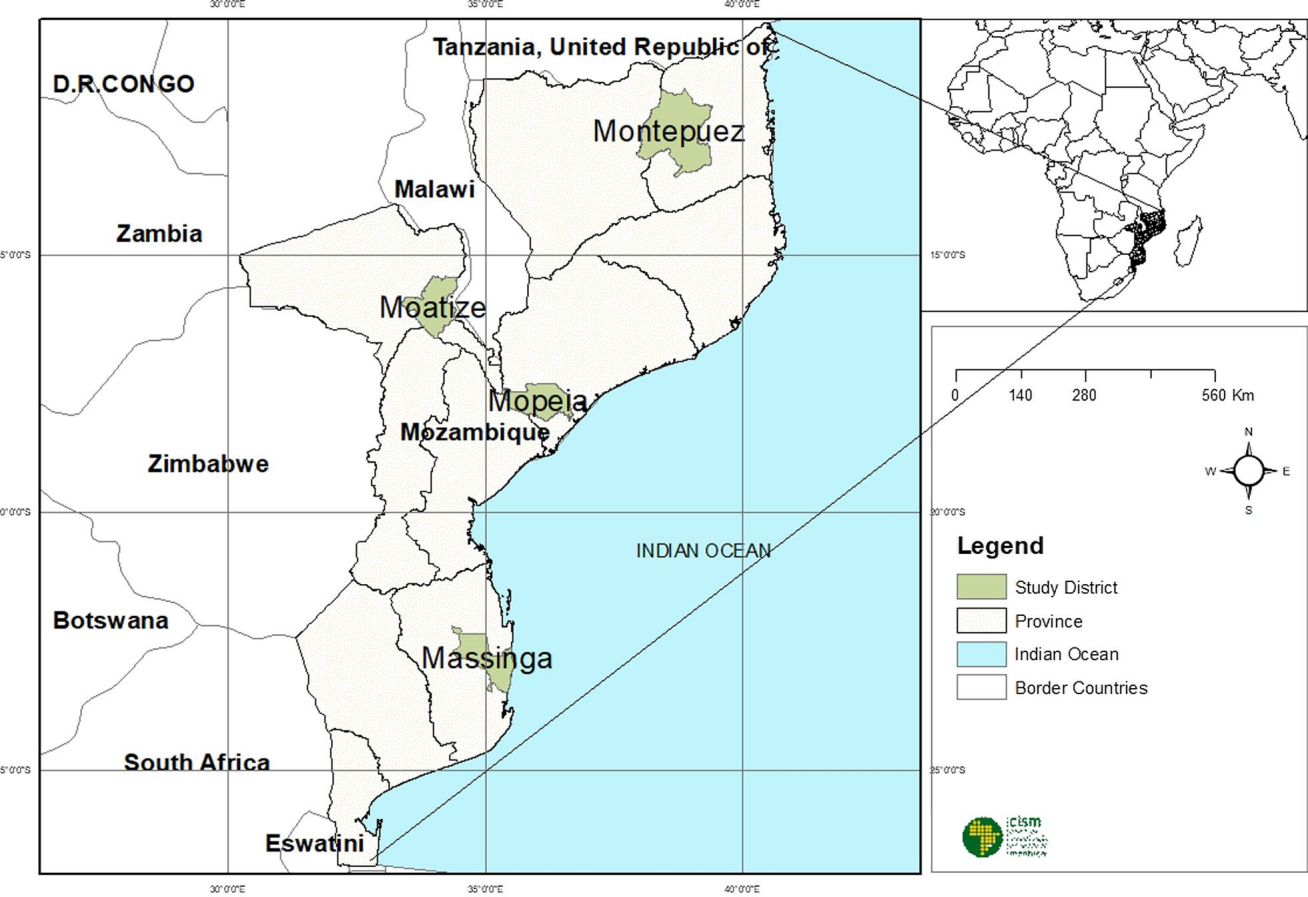
Location of sampling sites in Mozambique, 2018

This study was a sub-study of a TES that evaluated the efficacy and safety of AL and ASAQ in the treatment of uncomplicated *P. falciparum* malaria in children aged 6–59 months in Mozambique, based on WHO-recommended protocol [27]. Malaria transmission in the country is year-round, with seasonal peaks during and after the rainy season, which occurs between October and March. The peak of the malaria transmission extends from November into April [3]. This study was conducted between February and September 2018 in four sentinel sites: Rural Hospital of Montepuez, in Cabo Delgado Province (Northern region), Moatize Health Center, in Tete Province (Central region), District Hospital of Mopeia, in Zambézia (Central region), and District Hospital of Massinga, in Inhambane Province (Southern region) (Fig. 1). These sites are distributed across the Northern, Central, and Southern regions of Mozambique, which represent areas with high, moderate, and low prevalence of malaria, respectively. The per protocol PCR-corrected efficacy results of this study will be reported elsewhere, but were greater than 95% for all four AL arms and greater than 98% for all three ASAQ arms (no ASAQ arm in Moatize).

### Sample collection

Potential participants were screened for malaria parasites using microscopy at each study site. Patients were eligible for enrolment if they had uncomplicated *P. falciparum* mono-infection with an asexual blood density between 2000 and 200,000/µL, were aged 6–59 months, and had a fever at presentation (axillary temperature ≥ 37.5 °C) or history of fever in the last 24 h. A dried blood spot on Whatman 3-mm filter paper was prepared using 50 µL of blood collected on the day of enrolment (day 0/pre-treatment) and on any other day the patient had a recurrent malaria infection during the follow-up period (post-treatment).

### DNA extraction

DNA was extracted at the Manhiça Health Research Center Laboratory, Mozambique, from half of the dried blood spot using a QIAamp DNA Mini kit (QIAGEN, Hilden, Germany) according to the manufacturer’s instructions. The DNA was eluted in 150μL of elution buffer, aliquoted and transferred to the CDC Malaria Laboratory in Atlanta, GA, USA, for molecular analysis.

### Molecular genotyping of resistance markers

Molecular analysis for drug resistance markers was performed by a laboratory technician from Mozambique with the support of staff from the CDC Malaria Laboratory in Atlanta, USA [28]. For this analysis, selected pre-treatment and all post-treatment samples were used. Samples were first screened using a multiplex real-time PCR assay (PET-PCR) for detection of *Plasmodium* genus and *P. falciparum,* as previously described [29]. Polymorphisms in the *pfk13* (propeller domain 389-649), *pfcrt* (codons 72–76), and *pfmdr1* (codons 86, 184, 1034, 1042, and 1246) genes were investigated as previously described [30, 31]. Briefly, both pre-treatment and post-treatment samples were used to amplify fragments of *pfk13, pfcrt,* and *pfmdr1* by nested PCRs. Three laboratory *P. falciparum* parasite lines, 3D7, 7G8, and Dd2, were included as controls. Direct Sanger sequencing of the purified nested PCR products was performed using a BigDye Terminator v3.1 cycle sequencing kit on an iCycler thermal cycler (Bio-Rad, CA, USA). The reaction mixtures were precipitated in 70% ethanol to clean up dye terminators, rehydrated in 10 μL HiDi formamide, and then sequenced on a 3130xl ABI genetic analyzer (ABI Prism, CA, USA). Sequence analysis was performed using Geneious R7 (Biomatters, Auckland, New Zealand). Raw sequence reads were cleaned using default settings and reads with high-quality scores (> 30%) were further analysed using the 3D7 *pfk13, pfcrt,* and *pfmdr1* genes as references.

### Data analyses

Data were entered into a Microsoft Office Excel 2007 sheet and then exported into R 3.6.0 (R Core Team 2019) for validation, cleaning, and analysis. A statistical significance of difference in the risk of treatment failure (reinfection or recrudescence) was determined by Fisher’s exact test, at a 5% significance level. All possible haplotypes from mixed infections (both wild type and mutants) were included in construction of the *pfmdr1* haplotype.

## Results

### Characteristics of study subjects

**Fig. 2:**
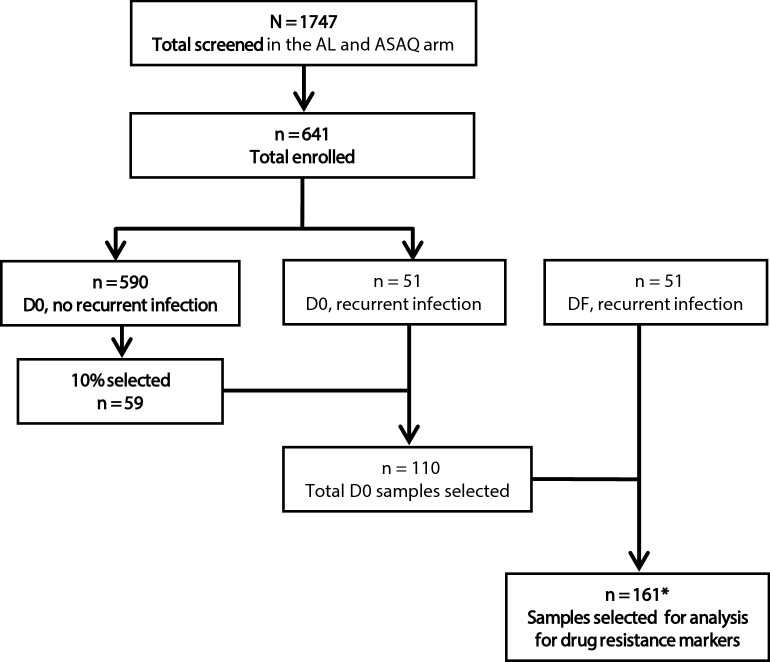
Selection of samples for analysis of molecular markers of resistance from 2018 TES samples. D0, day of enrolment; DF, day of recurrent infection; *a sample was not included due to poor quality DNA

From the 641 patients enrolled in the TES, 110 (17%) pre-treatment samples were selected for the analysis. This included all the pre-treatment samples from subjects who returned with a recurrent infection (n = 51) and 10% randomly selected pre-treatment samples from patients who did not have a recurrent infection (n = 59); however, one sample was excluded due to poor quality DNA, leaving 109 pre-treatment samples. All 51 post-treatment samples from patients who had a recurrent malaria infection were included in the analysis (Fig. 2). In the AL and ASAQ arms, 7.1% (26/368) and 3.0% (8/273) of subjects, respectively, remained parasitaemic at day 3, although no subject met criteria for early treatment failure.

Table 1 summarizes the characteristics by site of 109 study participants used for molecular analysis. A total of 79 and 30 pre-treatment samples, and 48 and 3 post-treatment samples, were in the AL and ASAQ treatment arms, respectively.

**Table 1:** Characteristics of study participants by site, Mozambique 2018

Number of subjects	Massinga	Moatize	Montepuez	Mopeia	Total
	39	12	20	38	109
Female sex n (%)	21 (53.8)	5 (41.7)	9 (45.0)	18 (47.4)	53 (48.6)
Age in months (mean ± SD)	28.5 ± 5.4	37.2 ± 16.3	21.8 ± 14.3	29.6 ± 14.4	28.6 ± 15.3
Temperature in °C (mean ± SD)	38.4 ± 0.8	38.7 ± 1.1	38 ± 0.8	37.9 ± 0.4	38.2 ± 0.8
Parasite density geometric mean (range)	28,100 (800–126,800)	52,900 (13,300–181,100)	30,500 (4800–107,500)	41,500 (4300–168,200)	35,100 (800–181,100)
Hb in g/dL (mean ± SD)	8.2 ± 1.7	9.5 ± 1.1	9.6 ± 1.9	9.4 ± 1.6	9 ± 1.7

SD, standard deviation; Hb, haemoglobin

### Molecular markers of drug resistance

#### *pfk13* polymorphisms

All 109 pre-treatment samples and 48/51 (94.1%) of the post-treatment samples were successfully sequenced at the *pfk13* gene. No polymorphisms associated with artemisinin partial resistance were observed in the propeller domain One sample from Mopeia contained a synonymous mutation at codon 469 (TGC to TGT) and three samples from Mopeia contained a synonymous mutation at codon 548 (GGC to GGT). No other synonymous or nonsynonymous mutations were found.

#### *pfcrt* polymorphisms

All 109 pre-treatment samples and 47/51 (92.2%) of the post-treatment samples were successfully sequenced at the *pfcrt* gene. All samples showed the wild type CVMNK haplotype.

#### *pfmdr1* polymorphisms

All 109 pre-treatment and 48/51 (94.1%) post-treatment samples were successfully sequenced for the *pfmdr1* gene. All pre-treatment samples possessed the N86, S1034, N1042 and D1246 alleles, with polymorphisms being observed only at codon 184: 184**F** in 43 (39.4%), Y184 in 42 (38.5%), and mixed Y/**F** in 24 (22.0%).

Among the 79 pre-treatment samples obtained from the AL arm, N**F**D (86, 184, 1246 codons) and NYD haplotypes were present in 49 (62.0%) and 45 (57.0%), respectively. In the ASAQ arm, N**F**D and NYD were present in 18 (60.0%) and 21 (70.0%) of the pre-treatment samples, respectively. *Neither N****F****D* nor NYD significantly changed (p > 0.05) in post-treatment infections after treatment with either AL or ASAQ (Table 2).

**Table 2:** Prevalence of *pfmdr1* 184 polymorphisms in pre-treatment and post-treatment samples stratified by treatment arms

*pfmdr1* Polymorphism	AL arm			ASAQ arm		
	Pre-treatment	Post-treatment	*p* value*	Pre-treatment	Post-treatment	*p* value*
	n (%)	n (%)		n (%)	n (%)	
*pfmdr1* codon 184	n = 79	n = 46		n = 30	n = 2	
Y184	30 (38.0)	12 (26.1)	Ref	12 (40.0)	0 (0)	Ref
184Y/**F**	15 (10.0)	16 (34.8)	0.054	9 (30.0)	1 (50.0)	0.454
184**F**	34 (43.0)	18 (39.1)	0.657	9 (30.0)	1 (50.0)	0.454
*pfmdr1* haplotypes^a^	n = 79	n = 46		n = 30	n = 2	
NYD	45 (57.0)	28 (60.9)	Ref	21 (70.0)	1 (50.0)	Ref
N**F**D	49 (62.0)	34 (73.9)	0.746	18 (60.0)	2 (100)	0.597

AL, artemether-lumefantrine; ASAQ, artesunate-amodiaquine

*Statistical significance in risk of recurrent infection (reinfection or recrudescence) was determined by Fisher’s exact test; three post-treatment samples (two in the AL arm and one in the ASAQ arm) failed to amplify at one or more loci and are not included in corresponding single nucleotide polymorphism and haplotype counts

^a^Haplotype percentages may not sum to 100% because all possible haplotypes from mixed infections (both wild type and mutants) were included in the construction of haplotypes

In the pre-treatment samples, N**F**D was present in 29 (74.4%) and 8 (66.7%) samples from Massinga and Moatize, respectively, while NYD was present in 15 (75.0%) and 26 (68.4%) samples from Montepuez and Mopeia, respectively. For the late treatment failure samples, the N**F**D was observed in all samples from Moatize and Montepuez and in 72.7% of the samples from Massinga and Mopeia (Table 3).

**Table 3:** Prevalence of *pfmdr1* polymorphisms in pre-treatment and post-treatment samples stratified by study site

*pfmdr1*^a^	Pre-treatment (N = 109)				Post-treatment (N = 48)			
	MEGA	MEZE	MEMP	MEIA	MEGA	MEZE	MEMP	MEIA
	n = 39	n = 12	n = 20	n = 38	n = 22	n = 1	n = 3	n = 22
Y184	10 (25.6)	4 (33.3)	9 (45.0)	19 (50.0)	6 (27.3)	0 (0)	0 (0)	6 (27.3)
184Y/**F**	8 (20.5)	3 (25.0)	6 (30.0)	7 (18.4)	8 (36.4)	1 (100)	0 (0)	8 (36.4)
184**F**	21 (53.8)	5 (41.7)	5 (25.0)	12 (31.6)	8 (36.4)	0 (0)	3 (100)	8 (36.4)
NYD	18 (46.2)	7 (58.3)	15 (75.0)	26 (68.4)	14 (63.6)	1 (100)	0 (0)	14 (63.6)
N**F**D	29 (74.4)	8 (66.7)	11 (55.0)	19 (50.0)	16 (72.7)	1 (100)	3 (100)	16 (72.7)

Samples are from both AL and ASAQ arms; MEGA: Massinga; MEZE: Moatize; MEMP: Montepuez; MEIA: Mopeia; three samples (two in the AL arm and one in the ASAQ arm) failed to amplify at day of failure at one or more loci and are not included in corresponding single nucleotide polymorphism and haplotype counts

^a^Percentages may not sum to 100% because all possible haplotypes from mixed infections (both wild type and mutants) were included in the construction of haplotypes. Tests of significance not performed due to low sample sizes in two sites

## Discussion

Mozambique has used AL and ASAQ as the two first-line anti-malarial regimens since 2009, with AL being the most widely used and ASAQ as backup for situations in which AL could not be used or is not available. This study, provides insights into the *pfk13, pfcrt,* and *pfmdr1* genetic profiles of *P. falciparum* isolates from four sentinel sites throughout the country. In this study, no *pfk13* mutations associated with artemisinin partial resistance were observed. These findings are encouraging and suggest that artemisinin partial resistance has not yet emerged in the four study sites selected in Mozambique. A previous study in Mozambique revealed a very low prevalence (< 1%) of four polymorphisms in the *pfk13* gene (L619L, F656I, V666V, and G690G)[17]. Another study revealed the presence of a V494I *K13* polymorphism, found in two samples collected after the introduction of ACT in Mozambique [32]; However, these aforementioned mutations are either synonymous or not known to be associated with artemisinin partial resistance [17]. This current study’s findings are also consistent with most reports from Africa in which no, or a very low prevalence of, *pfk13* mutations have been reported [33, 34]. The absence of delayed parasite clearance and *pfk13* mutations known to be associated with artemisinin partial resistance is reassuring for Mozambique, at least in the short term. Nevertheless, a recently published Rwandan study, using samples collected between 2012 and 2015, showed that 7.4% of the specimens carried the *pfk13* R561H mutation [15], known to be associated with artemisinin partial resistance. Another recent Rwandan study also detected the presence of two validated markers of artemisinin partial resistance, R561H and P574L, and delayed parasite clearance (parasitaemia at day 3) in more than 10% of the study participants in two sites [13]. Although this finding was not linked to clinical treatment failure of AL, it highlights the importance of conducting molecular surveillance to identify emerging patterns of parasites with artemisinin and partner drug resistance genotypes.

The *pfcrt* data from this study showed that all samples sequenced contained the wild type *pfcrt* haplotype (CVMNK), suggesting the return of chloroquine sensitive alleles after its use was discontinued in 2003 in Mozambique. This finding is consistent with data from previous studies carried out in Mozambique that reported an increasing frequency of the *pfcrt* CVMNK wild type, from 43.9 to 66.4% between 2009 and 2010 [22]. A Mozambique study from 2015 reported a very low prevalence of mutant alleles at codons M74I, N75E, and K76T; only 2.3% samples harbored the *pfcrt* SNP 76**T** [17]. A separate report from 2015 reported only 0.9% samples with the *pfcrt* 76**T** mutant allele, 3.7% samples with a mixed infection, and 95.4% samples with the wild type allele [35]. These findings confirm the likely return of CQ-susceptible *P. falciparum* and are similar to findings from studies conducted in other African countries that also observed a resurgence in the proportion of wild type *pfcrt* alleles after the discontinuation of CQ for treatment [36–38]. AL has been shown to select for *pfcrt* wild types [22, 39], and the widespread use of AL in most African countries may contribute to the re-emergence of these alleles associated with CQ sensitivity [39].

Polymorphisms in *pfmdr1* were only observed at codon 184, resulting in two observed haplotypes, N**F**D and NYD. This is consistent with previous findings from Mozambique. In 2015, a low prevalence of 86**Y** (3.1%) and a higher prevalence of 184**F** (46.7%) were reported [17]. In addition, a high prevalence of wild type N86 (73.2%) and D1246 (96.7%) and the presence of the mutant 184**F** (22.7%) were reported in a 2010–2012 study [8].

The *pfmdr1* gene has been implicated in lower sensitivity or tolerance to several anti-malarial drugs, including lumefantrine, CQ, and AQ [22, 24]*,* with the 86**Y** mutation being associated with decreased CQ and AQ sensitivity and the N86 wild type allele implicated in decreased sensitivity to lumefantrine [25]. The N**F**D haplotype increased in prevalence between the pre- and post-treatment samples in this study’s AL arm, but this was not significant when compared with NYD. In Mozambique, NFD haplotype prevalence increased from approximately 22–38% between 2009 and 2010 [22]. While the sites from this study are not comparable to that report, the 61.5% pre-treatment NFD prevalence indicates that this haplotype is still circulating. Similar findings over time have been reported in other African countries in which AL was used as the first-line anti-malarial treatment [40, 41]. Some studies showed that the *pfmdr1* gene polymorphism at codons N86Y, Y184F, and D1246Y is mainly linked to AL or ASAQ drug pressure [42, 43]. Stratifying by site, N**F**D was identified in 100% of post-treatment samples from Moatize and Montepuez and 72.7% of samples from Massinga and Mopeia, although this was not statistically significant. Notable limitations of this study include a low sample size in some sites, due to few late recurrences, and the limited number of pre-treatment samples analysed, due to budgetary restrictions.

## Conclusion

Given that no *pfk13* or *pfcrt* molecular markers of resistance were observed, the results of this study corroborate the findings of the associated TES that showed AL and ASAQ were efficacious. The high prevalence of the *pfmdr1* N**F**D haplotype, associated with decreased sensitivity to lumefantrine in some studies, requires further investigation to fully understand the role of this haplotype in the sensitivity of the currently used artemisinin-based combinations, AL and ASAQ. Because alleles associated with artemisinin partial resistance are emerging in the East Africa region, continued molecular surveillance for early detection of these alleles as well as relevant partner drug resistant markers remains important.

## Data Availability

The datasets generated during and/or analysed during the current study are available from the corresponding author on reasonable request.

## Abbreviations

ACT: Artemisinin-based combination therapy
AL: Artemether-lumefantrine
AQ: Amodiaquine
AS: Artesunate
ASAQ: Artesunate-amodiaquine
CDC: Centers for Disease Control and Prevention
CIBERESP: Consorcio de Investigación Biomédica en Red de Epidemiología y Salud (Consortium for Biomedical Research in Epidemiology and Public Health)
CISM: Centro de Investigação em Saúde de Manhiça (Manhiça Health Research Centre)
CQ: Chloroquine
INS: Instituto Nacional de Saúde (Mozambican National Institute of Health)
PCR: Polymerase chain reaction
*pfcrt*: *Plasmodium falciparum* *chloroquine resistance transporter*
*pfk13*: *Plasmodium falciparum* *Kelch 13*
*pfmdr1*: *Plasmodium falciparum* *multidrug resistance 1*
SNP: Single nucleotide polymorphism
SP: Sulfadoxine-pyrimethamine
WHO: World Health Organization

## References

[1] WHO. World malaria report 2020: 20 years of global progress and challenges. Geneva, World Health Organization. 2020. https://apps.who.int/iris/handle/10665/337660. Accessed May 2021.

[2] National Malaria Control Programme (NMCP). 2017–2022 National malaria control strategic plan. Mozambique. 2017. http://pdf.usaid.gov. Accessed May 2021.

[3] President’s Malaria Initiative (PMI). Evaluation of the impact of malaria control interventions on all-cause mortality in children under five years of age in Mozambique—Mozambique Malaria Impact Evaluation Group. 2016; p. 155. http://pdf.usaid.gov. Accessed May 2021.

[4] WHO. Guidelines for malaria. Geneva, World Health Organization. 2021. https://www.who.int/publications/i/item/guidelines-for-malaria. Accessed May 2021.

[5] Abacassamo F, Enosse S, Aponte JJ, Gómez-Olivé FX, Quintó L, Mabunda S (2004). Efficacy of chloroquine, amodiaquine, sulphadoxine-pyrimethamine and combination therapy with artesunate in Mozambican children with non-complicated malaria. Trop Med Int Health.

[6] Nhama A, Bassat Q, Enosse S, Nhacolo A, Mutemba R, Carvalho E (2014). In vivo efficacy of artemether-lumefantrine and artesunate-amodiaquine for the treatment of uncomplicated falciparum malaria in children: a multisite, open-label, two-cohort, clinical trial in Mozambique. Malar J.

[7] Salvador C, Rafael B, Matsinhe F, Candrinho B, Muthemba R, De Carvalho E (2017). Efficacy and safety of artemether–lumefantrine for the treatment of uncomplicated falciparum malaria at sentinel sites in Mozambique, 2015. Acta Trop.

[8] Lobo E, De Sousa B, Rosa S, Figueiredo P, Lobo L, Pateira S (2014). Prevalence of pfmdr1 alleles associated with artemether-lumefantrine tolerance/resistance in Maputo before and after the implementation of artemisinin-based combination therapy. Malar J.

[9] Ouji M, Augereau JM, Paloque L, Benoit-Vical F (2018). *Plasmodium falciparum* resistance to artemisinin-based combination therapies: a sword of Damocles in the path toward malaria elimination. Parasite.

[10] Ariey F, Witkowski B, Amaratunga C, Beghain J, Ma L, Lim P (2016). A molecular marker of artemisinin-resistant *Plasmodium falciparum* malaria. Nature.

[11] Phyo AP, Nkhoma S, Stepniewska K, Ashley EA, Nair S, McGready R (2012). Emergence of artemisinin-resistant malaria on the western border of Thailand: a longitudinal study. Lancet.

[12] Amaratunga C, Lim P, Suon S, Sreng S, Mao S, Sopha C (2017). Dihydroartemisinin–piperaquine resistance in *Plasmodium falciparum* malaria in Cambodia: a multisite prospective cohort study. Lancet Infect Dis.

[13] Uwimana A, Umulisa N, Venkatesan M, Svigel SS, Zhou Z, Munyaneza T (2021). Association of *Plasmodium falciparum* kelch13 R561H genotypes with delayed parasite clearance in Rwanda: an open-label, single-arm, multicentre, therapeutic efficacy study. Lancet Infect Dis.

[14] WHO. Report on antimalarial drug efficacy, resistance and response 10 years of surveillance (2010–2019). Geneva, World Health Organization. 2020. https://www.who.int/publications/i/item/9789240012813. Accessed May 2021.

[15] Uwimana A, Legrand E, Stokes BH, Ndikumana JLM, Warsame M, Umulisa N (2020). Emergence and clonal expansion of in vitro artemisinin-resistant *Plasmodium falciparum* kelch13 R561H mutant parasites in Rwanda. Nat Med.

[16] Bergmann C, Van LW, Habarugira F, Tacoli C, Jäger JC, Savelsberg D (2021). Increase in kelch 13 polymorphisms in *Plasmodium falciparum*, Southern Rwanda. Emerg Infect Dis.

[17] Gupta H, Macete E, Bulo H, Salvador C, Warsame M, Carvalho E (2018). Drug-resistant polymorphisms and copy numbers in *Plasmodium falciparum*, Mozambique, 2015. Emerg Infect Dis.

[18] Gupta H, Galatas B, Chidimatembue A, Huijben S, Cisteró P, Matambisso G (2020). Effect of mass dihydroartemisinin–piperaquine administration in southern Mozambique on the carriage of molecular markers of antimalarial resistance. PLoS ONE.

[19] Holmgren G, Gil JP, Ferreira PM, Veiga MI, Obonyo CO, Björkman A (2006). Amodiaquine resistant *Plasmodium falciparum* malaria in vivo is associated with selection of pfcrt 76T and pfmdr1 86Y. Infect Genet Evol.

[20] Happi CT, Gbotosho GO, Folarin OA, Bolaji OM, Sowunmi A, Kyle DE (2006). Association between mutations in *Plasmodium falciparum* chloroquine resistance transporter and *P. falciparum* multidrug resistance 1 genes and in vivo amodiaquine resistance in *P. falciparum* malaria-infected children in Nigeria. Am J Trop Med Hyg.

[21] Djimdé A, Doumbo O, Cortese J, Kayentao K, Doumbo S, Diourté Y (2001). A molecular marker for chloroquine-resistant falciparum malaria. N Engl J Med.

[22] Thomsen TT, Madsen LB, Hansson HH, Tomás EVE, Charlwood D, Bygbjerg IC (2013). Rapid selection of *Plasmodium falciparum* chloroquine resistance transporter gene and multidrug resistance gene-1 haplotypes associated with past chloroquine and present artemether-lumefantrine use in Inhambane District, southern Mozambique. Am J Trop Med Hyg.

[23] Gadalla NB, Tavera G, Mu J, Kabyemela ER, Fried M, Duffy PE (2015). Prevalence of *Plasmodium falciparum* anti-malarial resistance-associated polymorphisms in pfcrt, pfmdr1 and pfnhe1 in Muheza, Tanzania, prior to introduction of artemisinin combination therapy. Malar J.

[24] Huijben S, Macete E, Mombo-Ngoma G, Ramharter M, Kariuki S, Desai M (2020). Counter-selection of antimalarial resistance polymorphisms by intermittent preventive treatment in pregnancy. J Infect Dis.

[25] Venkatesan M, Gadalla NB, Stepniewska K, Dahal P, Nsanzabana C, Moriera C (2014). Polymorphisms in Plasmodium falciparum chloroquine resistance transporter and multidrug resistance 1 genes: parasite risk factors that affect treatment outcomes for *P. falciparum* malaria after artemether-lumefantrine and artesunate-amodiaquine. Am J Trop Med Hyg.

[26] Nguetse CN, Adegnika AA, Agbenyega T, Ogutu BR, Krishna S, Kremsner PG (2017). Molecular markers of anti-malarial drug resistance in Central, West and East African children with severe malaria. Malar J.

[27] WHO. Methods for surveillance of antimalarial drug efficacy. Geneva, World Health Organization. 2009. https://www.who.int/docs/default-source/documents/publications/gmp/methods-for-surveillance-of-antimalarial-drug-efficacy.pdf?sfvrsn=29076702_2. Accessed May 2021.

[28] Halsey ES, Venkatesan M, Plucinski MM, Talundzic E, Lucchi NW, Zhou Z (2017). Capacity development through the US President’s malaria initiative-supported antimalarial resistance monitoring in Africa network. Emerg Infect Dis.

[29] Lucchi NW, Narayanan J, Karell MA, Xayavong M, Kariuki S, DaSilva AJ (2013). Molecular diagnosis of malaria by photo-induced electron transfer fluorogenic primers: PET-PCR. PLoS ONE.

[30] Vinayak S, Alam T, Sem R, Shah N, Susanti A, Lim P (2010). Multiple genetic backgrounds of the amplified *Plasmodium falciparum* multidrug resistance (pfmdr1) gene and selective sweep of 184F mutation in Cambodia. J Infect Dis.

[31] Talundzic E, Chenet SM, Goldman IF, Patel DS, Nelson JA, Plucinski MM (2015). Genetic analysis and species specific amplification of the artemisinin resistance-associated kelch propeller domain in *P. falciparum* and *P. vivax*. PLoS ONE.

[32] Escobar C, Pateira S, Lobo E, Lobo L, Teodosio R, Dias F (2015). Polymorphisms in *Plasmodium falciparum* K13-propeller in angola and mozambique after the introduction of the ACTs. PLoS ONE.

[33] Somé AF, Sorgho H, Zongo I, Bazié T, Nikiéma F, Sawadogo A (2016). Polymorphisms in K13, pfcrt, pfmdr1, pfdhfr, and pfdhps in parasites isolated from symptomatic malaria patients in Burkina Faso. Parasite.

[34] Matrevi SA, Opoku-Agyeman P, Quashie NB, Bruku S, Abuaku B, Koram KA (2019). *Plasmodium falciparum* kelch propeller polymorphisms in clinical isolates from Ghana from 2007 to 2016. Antimicrob Agents Chemother.

[35] Galatas B, Nhamussua L, Candrinho B, Mabote L, Cisteró P, Gupta H (2017). In-vivo efficacy of chloroquine to clear asymptomatic infections in Mozambican adults: a randomized, placebo-controlled trial with implications for elimination strategies. Sci Rep.

[36] Bwire GM, Ngasala B, Mikomangwa WP, Kilonzi M, Kamuhabwa AAR (2020). Detection of mutations associated with artemisinin resistance at k13-propeller gene and a near complete return of chloroquine susceptible falciparum malaria in Southeast of Tanzania. Sci Rep.

[37] Mwanza S, Joshi S, Nambozi M, Chileshe J, Malunga P, Kabuya JBB (2016). The return of chloroquine-susceptible *Plasmodium falciparum* malaria in Zambia. Malar J.

[38] Frosch AEP, Laufer MK, Mathanga DP, Takala-Harrison S, Skarbinski J, Claassen CW (2014). Return of widespread chloroquine-sensitive *Plasmodium falciparum* to Malawi. J Infect Dis.

[39] Sisowath C, Petersen I, Veiga MI, Mårtensson A, Premji Z, Björkma A (2009). In vivo selection of *Plasmodium falciparum* parasites carrying the chloroquine-susceptible pfcrt K76 allele after treatment with artemether-lumefantrine in Africa. J Infect Dis.

[40] Okell LC, Reiter LM, Ebbe LS, Baraka V, Bisanzio D, Watson OJ (2018). Emerging implications of policies on malaria treatment: genetic changes in the Pfmdr-1 gene affecting susceptibility to artemether–lumefantrine and artesunate–amodiaquine in Africa. BMJ Glob Health.

[41] Ishengoma DS, Mandara CI, Francis F, Talundzic E, Lucchi NW, Ngasala B (2019). Efficacy and safety of artemether-lumefantrine for the treatment of uncomplicated malaria and prevalence of Pfk13 and Pfmdr1 polymorphisms after a decade of using artemisinin-based combination therapy in mainland Tanzania. Malar J.

[42] Baliraine FN, Rosenthal PJ (2011). Prolonged selection of pfmdr1 polymorphisms after treatment of falciparum malaria with artemether-lumefantrine in Uganda. J Infect Dis.

[43] Okombo J, Kamau AW, Marsh K, Sutherland CJ, Ochola-Oyier LI (2014). Temporal trends in prevalence of *Plasmodium falciparum* drug resistance alleles over two decades of changing antimalarial policy in coastal Kenya. Int J Parasitol Drugs Drug Resist.

